# A role for the *Drosophila* zinc transporter *Zip88E* in protecting against dietary zinc toxicity

**DOI:** 10.1371/journal.pone.0181237

**Published:** 2017-07-13

**Authors:** Christopher D. Richards, Coral G. Warr, Richard Burke

**Affiliations:** School of Biological Sciences, Monash University, Victoria, Australia; CINVESTAV-IPN, MEXICO

## Abstract

Zinc absorption in animals is thought to be regulated in a local, cell autonomous manner with intestinal cells responding to dietary zinc content. The *Drosophila* zinc transporter Zip88E shows strong sequence similarity to Zips 42C.1, 42C.2 and 89B as well as mammalian Zips 1, 2 and 3, suggesting that it may act in concert with the apically-localised *Drosophila* zinc uptake transporters to facilitate dietary zinc absorption by importing ions into the midgut enterocytes. However, the functional characterisation of *Zip88E* presented here indicates that *Zip88E* may instead play a role in detecting and responding to zinc toxicity. Larvae homozygous for a null *Zip88E* allele are viable yet display heightened sensitivity to elevated levels of dietary zinc. This decreased zinc tolerance is accompanied by an overall decrease in *Metallothionein B* transcription throughout the larval midgut. A *Zip88E* reporter gene is expressed only in the salivary glands, a handful of enteroendocrine cells at the boundary between the anterior and middle midgut regions, and in two parallel strips of sensory cell projections connecting to the larval ventral ganglion. *Zip88E* expression solely in this restricted subset of cells is sufficient to rescue the *Zip88E* mutant phenotype. Together, our data suggest that *Zip88E* may be functioning in a small subset of cells to detect excessive zinc levels and induce a systemic response to reduce dietary zinc absorption and hence protect against toxicity.

## Introduction

Zinc is an essential dietary nutrient, required as a structural or enzymatic cofactor for potentially thousands of different proteins. It has been estimated that up to 10% of all human proteins are able to bind zinc [1]. There is also a growing body of evidence that unbound zinc ions may be able to act as signalling molecules to regulate cellular processes such as growth and neurotransmission [2].

Movement of zinc ions across cell membranes is facilitated by two large classes of proteins. Members of the Zip family have mostly been shown to transport zinc into the cytosol, either from outside the cell (cellular uptake) or from the lumen of cellular organelles in order to redistribute zinc within individual cells. ZnT proteins mostly function in the opposite direction, removing zinc from the cell (cellular efflux) or supplying organelles such as the endoplasmic reticulum, Golgi and lysosome. Within such organelles, zinc may be loaded onto proteins that require zinc for their activity, stored for later use, or packaged for removal. The large number of Zip and ZnT proteins encoded by vertebrate and invertebrate genomes indicates that each protein has taken on a specialised role defined in part by its expression pattern, cellular localisation, zinc transport ability / specificity and post-translational regulation.

The relative simplicity of the zinc transport network in *Drosophila* (17 *Zip* and *ZnT* genes compared to 25 in vertebrates [3]) has facilitated the functional characterisation of a number of these genes. Focussing on the process of zinc absorption from the diet, two Zip proteins, Zip42C.1 and Zip42C.2, have been shown to play a major role in uptake of zinc from the intestinal lumen through a small cluster of cells in the larval midgut called the iron cells [4]. A third, closely related protein, Zip89B, is more widely expressed throughout the midgut and appears to play an ancillary role in zinc absorption [5]. Although *Zip89B* is non-essential, in its absence, *Zip42C*.*1* and *Zip42C*.*2* are upregulated, presumably to compensate for a reduction in zinc uptake [5]. Once inside the cells of the fly midgut, zinc must be released into the circulatory system for systemic supply, a function performed by *ZnT63C* with support from *ZnT77C* [4, 6].

Together with a fourth protein, Zip88E, Zips 89B, 42C.1 and 42C.2 form a highly-conserved clade with highest similarity to mammalian Zips 1, 2 and 3 [3]. Indeed, this is the only situation where there are more fly proteins than human proteins within a Zip or ZnT phylogenetic subgroup. The *Zip1* to *3* mouse triple knockout shows no obvious defects in mice raised on a zinc replete diet [7]. The *Zip1* and *Zip3* single knockout mice do however show a high level of embryonic developmental abnormalities in pups of knockout mice raised on a zinc-deficient diet and in the *Zip1/3* double knockout mice these defects are elevated in an additive fashion. The *Zip1/2/3* triple knockout mouse has zinc deficiency phenotypes equivalent to those of the *1/3* double knockout suggesting that while *Zip1* and *Zip3* are playing overlapping roles, *Zip2* functions differently. Indeed, expression of *Zips 1* and *3* is broad and includes the intestinal stromal cells whereas *Zip2* expression is limited to the pericentral hepatocytes, keratinocytes and immature dendritic cells [8].

*Zip4* has been considered as the major zinc uptake gene in the mammalian intestine to date because a human zinc deficiency disease, Acrodermatitis enteropathica, is caused by *Zip4* mutations [9]. However, intestinal-specific knockout of *Zip4* actually results in a reprogramming of Paneth cells, accompanied by crypt dysplasia and reduced cell division in the small intestine [10]. Therefore the systemic zinc shortage caused by *Zip4* mutations may in fact be due to general intestinal malfunction resulting from zinc deficiency specifically in the Paneth cells. In this scenario, *Zips 1* and *3* may play an important role in general zinc absorption via the enterocytes, although clearly *Zip4* and / or other uptake mechanisms must also be contributing since the *Zip1/3* double knockout is relatively healthy.

While *Drosophila Zips 89B*, *42C*.*1* and *42C*.*2* have been well characterized, the closely-related *Zip88E* is yet to be examined in detail. Previous over-expression experiments have shown that Zip88E is localized both to the outer basolateral membrane and an endomembrane but does not overlap with endoplasmic reticulum or Golgi markers [11]. *Zip88E* overexpression alone has no effect on *Drosophila* viability or morphology but does modify over expression phenotypes of other fly *Zip* and *ZnT* genes. In these interaction experiments, *Zip88E* acts to increase cytosolic zinc levels, behaving similarly to *Zips 89B*, *42C*.*1* and *42C*.*1* but even more like the uncharacterised *Zips 102B* and *99C* [11].

Double knockdown of *Zips 42C*.*1* and *42C*.*2* in the fly larval midgut clearly causes zinc deficiency in animals raised on a zinc-poor diet yet has relatively little effect on a zinc-replete diet [4], indicating that additional enterocyte uptake mechanisms may be contributing to zinc absorption in the fly. A similar phenomenon is observed in mouse *Zip* gene knockouts, suggesting that alternative zinc absorption mechanisms could be novel therapeutic targets for addressing zinc deficiency. Here we have generated a null mutation in *Zip88E* and examined its expression pattern to investigate whether this closely-related gene may be playing a supporting role in zinc absorption.

## Materials and methods

### *Drosophila* stocks

The following fly stocks were used: *w*^*1118*^ (BL3605, Bloomington Stock Centre, Indiana USA); *GMR-GAL4* (*P[longGMR-GAL4]3*, BL8121). RNA interference (RNAi) lines were obtained from the Vienna *Drosophila* RNAi Centre (VDRC)). *MtnB*:*EYFP* was a gift from Walter Schaffner (University of Zurich, Switzerland). Microscopy utilized *P[UAS-mCD8*::*GFP*.*L]2* and *P[UAS-2xEGFP]AH2* to visualize reporter gene expression. A list containing the transgenic lines used in this study is provided in [11]. All transgenic *Drosophila* experiments carried out in this research were performed with the approval of the Monash University Institutional Biosafety Committee. No ethics approval is required for experiments involving insects in Australia.

### Cloning and generation of *Zip88E*:*GAL4* reporter construct

The predicted promoter/enhancer regions of *Zip88E* were PCR-amplified from genomic DNA extracted from *w*^*1118*^ third instar larvae. The region directly upstream of the START codon of *Zip88E* until the STOP codon of the preceding gene (*CG14864*) was amplified and cloned into a modified *pUAST-attB* vector with the UAS sequence upstream of the multiple cloning site (MCS) removed. Full length *GAL4* coding sequence was also cloned into the MCS. This construct was injected into *PhiC31 attP 51C* and *86Fb* strains (provided by Konrad Basler). Microinjections utilized an Eppendorf Femtojet apparatus with Femtotips II pre-pulled glass needles (Eppendorf). Oligonucleotide sequences are provided in S1 Table.

### *Drosophila* maintenance and feeding experiments

All *Drosophila* strains and crosses were maintained on standard (basal) medium at 25°C unless stated otherwise. Standard medium was supplemented with 4–12 mmol l^-1^ zinc chloride (ZnCl_2_; Sigma Aldrich, St. Louis, MO, USA) to make zinc-supplemented medium, or 50–150 μmol l^-1^ N,N,N’,N’-tetrakis (2-pyridylmethyl)-ethylenedidiamine (TPEN; Sigma Aldrich) to make zinc-deficient medium. For survival assays, *Drosophila* first instar larvae were transferred between 20–24 h post-emergence onto media supplemented with aqueous pre-diluted ZnCl_2_ or TPEN (50 larvae per replicate). Adult survival was determined as the proportion of larvae that had emerged as adults after 15 days at 25°C. Acute exposure to supplemented food was achieved by picking third instar larvae before the wandering stage onto treated food types and allowing development for 20–24 h before further analysis was conducted.

### Imprecise P-element excision

Males with the P-element *P[EPgy2]Zip88E*^*EY11179*^ (BL20270) inserted just upstream of the *Zip88E* translation START codon were crossed to the *Δ2*,*3 (99B)* transposase stock to induce an imprecise excision event. Single male progeny with mosaic eyes were crossed to *MRKS/TM6β* females. Single white-eyed males in the subsequent generation were then tested for imprecise excision events, using PCR primers designed to span the majority of the *Zip88E* locus. Oligonucleotide sequences are provided in S1 Table.

### Microscopy

Adult flies were partially dissected then mounted directly onto plasticine and monitored with a Leica MZ6 stereomicroscope. All eye images were recorded on a Leica DC300n digital camera and Leica Application Suite.

Midguts, salivary glands and the brain were dissected from third instar larvae in cold phosphate buffered saline (PBS) and mounted directly onto glass slides in Vectorshield (Vectorlabs) or fixed in 4% paraformaldehyde in PBS. Monoclonal α-Prospero and α–Fasciclin I primary antibodies used for Zip88E>mCD8::eGFP co-localization studies were obtained from the Developmental Studies Hybridoma Bank and used at 1:50 and 1:200 dilution respectively, followed by α-mouse AlexaFluor568 secondary antibody (Molecular Probes) used at 1:1000 dilution. MtnB:EYFP fluorescence in larval tissue was viewed on a Leica M165 FC dissecting microscope using a Leica DFC450 camera and Leica Application Suite. Higher magnification imaging was performed on either: 1) a Leica DMLB compound microscope using a Leica DC300 camera and Leica Application Suite at a magnification of 10x and 20x; or 2) an Olympus CV1000 spinning disk confocal microscope with a 10x dry objective lens or a 60x immersion objective lens.

### Western blot analysis

Extraction of protein lysate was achieved by homogenising five whole third instar larvae in 2% SDS lysis buffer with a protease inhibitor cocktail (Sigma Aldrich). Protein samples were resolved on 4–12% NuPAGE^®^ Bis-Tris gels (Invitrogen) and were transferred to a poly-vinylidene di-fluoride membrane (Milipore) using the X Cell Surelock^™^ Mini Cell system (Invitrogen). Ponceau S (Sigma) staining was used to assess efficiency of the transfer and provide confirmation of equal sample loading between lanes. α-GFP (rabbit, Molecular Probes) primary antibody was used at 1:10,000 in 5% skim milk solution. Blots were viewed using the QUANTUM ST5 Gel Documentation System (Vilber Lourmat).

### Semi-quantitative RT-PCR analysis

RNA extraction was performed by homogenising midguts in TRIsure RNA reagent (Bioline). Reverse transcription was performed using Tetro cDNA Synthesis Kit (Bioline). Semi-quantitative PCR analysis was performed using GoTaq green master mix with 1 μl of 100 ng/ μl cDNA used per reaction. PCR products were separated by electrophoresis on a 2.5% agarose gel. Housekeeping gene *RP49* was used as an endogenous control. Oligonucleotide sequences are provided in S1 Table.

### Statistical analysis

Two-way ANOVA analysis followed by Tukey multiple comparisons test and multiple T-test analysis followed by the Holm-Sidak multiple comparison test were performed using GraphPad Prism version 6.00 for Windows, GraphPad Software, La Jolla California USA, www.graphpad.com. Statistical significance was deemed when the p-value ≤ 0.05. Quantification of western blot band intensity was achieved using Image J analysis software (1.47v).

## Results

**Fig 1:**
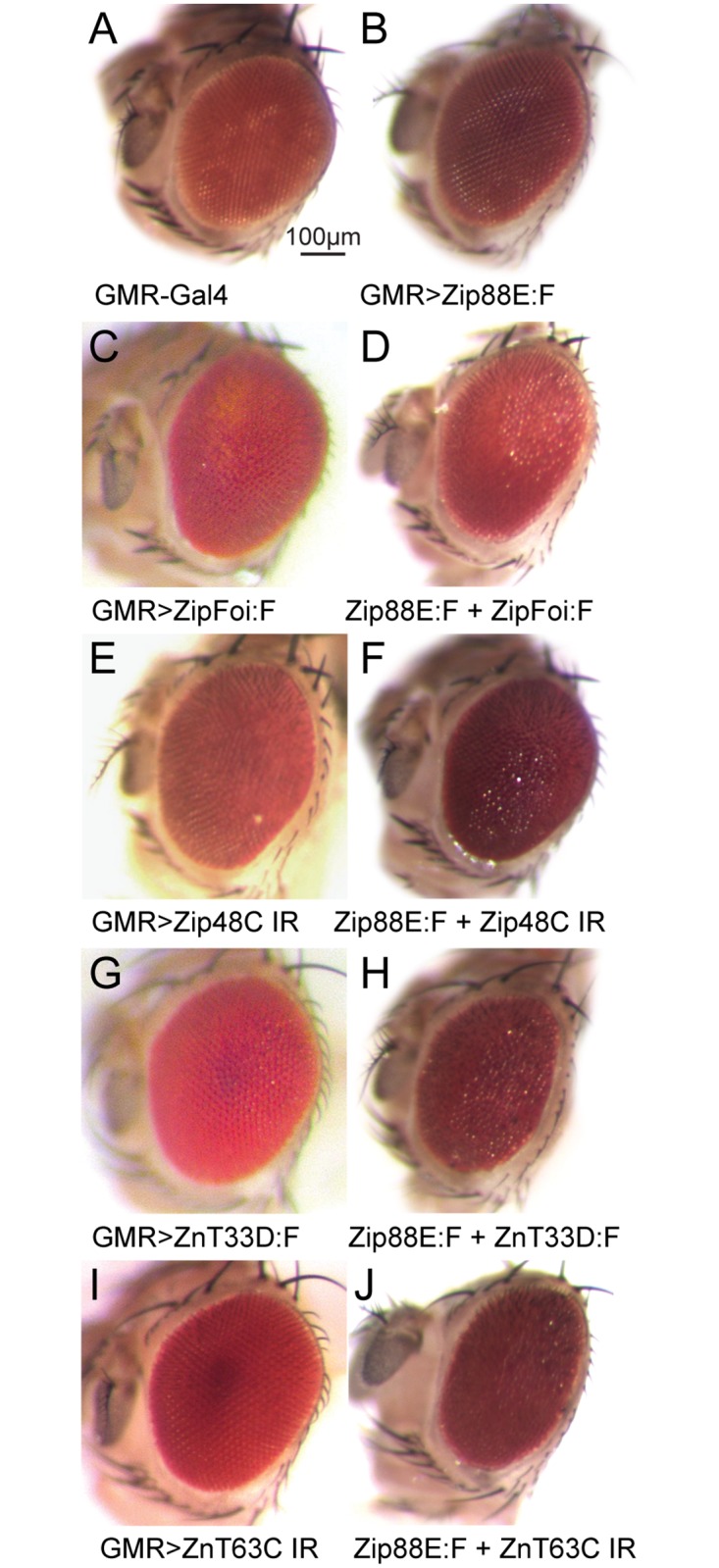
*Zip88E* over expression interacts with other zinc transport gene manipulations to disrupt eye development. *GMR-GAL4* was used to drive ectopic *Zip88E*::*FLAG* expression in the developing *Drosophila* eye, alone and in combination with the over expression and RNAi (IR) knockdown of various *Zip* and *ZnT* genes. A) *GMR-GAL4-*only control. B) *GMR>Zip88E*::*FLAG*. C) *GMR>ZipFoi*::*FLAG*. D) *GMR>Zip88E*::*FLAG + ZipFoi*::*FLAG*. E) *GMR>Zip48C IR*. F) *GMR>Zip88E*::*FLAG + Zip48C IR*. G) *GMR>ZnT33D*::*FLAG*. H) *GMR>Zip88E*::*FLAG + ZnT33D*::*FLAG*. I) *GMR>ZnT63C IR*. J) *GMR>Zip88E*::*FLAG + ZnT63C IR*.

Previous, limited functional characterisation of *Zip88E* by targeted over expression and RNAi-mediated knockdown indicated that it acts to increase cytosolic zinc levels; over expression in the adult eye exacerbated zinc toxicity phenotypes caused by *Zip71B*::*FLAG* and *ZnT86D* over expression while *Zip88E* knockdown had the opposite effect, rescuing both these phenotypes back to wild type [11]. This analysis was extended by examining interactions between over expression of *Zip88E*::*FLAG* and manipulation of all other *Drosophila Zip* and *ZnT* genes, using *GMR-GAL4* to drive expression only in the eye. While over expression of *Zip88E*::*FLAG* alone had no impact on eye morphology (Fig 1B), co-expression with *ZipFoi*::*FLAG* (Fig 1D*)*, *Zip48C IR* (Fig 1F), *ZnT33D*::*FLAG* (Fig 1H), and *ZnT63C IR* (Fig 1J) all caused mild but detectable disruptions to eye morphology. All other *Zip* / *ZnT* transgenes caused no appreciable phenotype when co-expressed with *Zip88E*::*FLAG* (S1 Fig).

To further characterise the systemic role of *Zip88E* during fly development, a putative null mutation was generated by imprecise P-element excision, creating a deletion removing the START codon and all of the first and second introns encoding the first 170 amino acids of the protein (S2 Fig). No *Zip88E* transcript could be amplified from cDNA extracted from *Zip88E*^*Δ/Δ*^ homozygous larvae (S3 Fig), supporting the proposition that this deletion constitutes a null allele for *Zip88E*.

**Fig 2:**
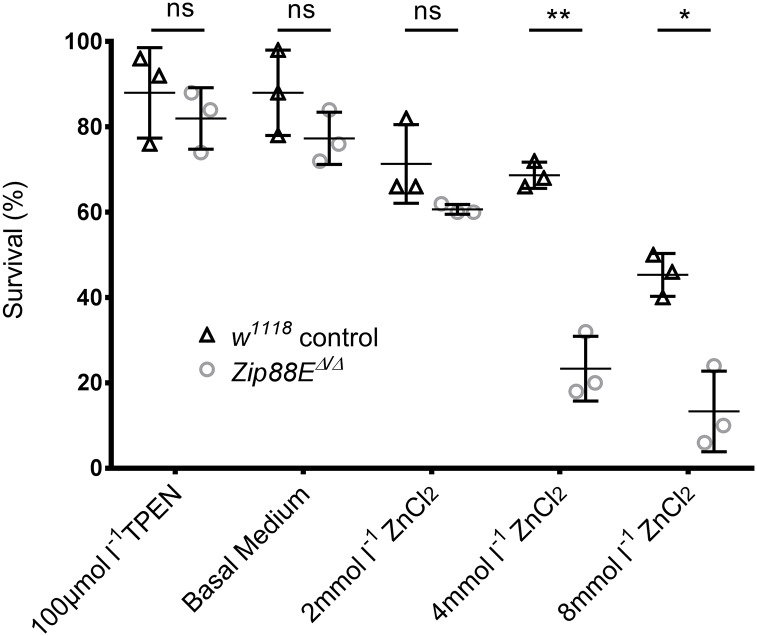
Larvae lacking *Zip88E* have increased susceptibility to high dietary zinc. Mean survival rates of control (*w*^*1118*^) and *Zip88E*^*Δ/Δ*^ flies raised from first instar larvae on zinc-supplemented and zinc-chelated media. There is no effect on survival of *Zip88E*^*Δ/Δ*^ flies on basal, zinc-chelated (TPEN) and 2 mmol l^-1^ ZnCl_2_-supplemented media compared to control flies. *Zip88E*^*Δ/Δ*^ flies have a significantly decreased survival rate on 4 and 8 mmol l^-1^ ZnCl_2_-supplemented media compared to control flies (values are represented as the mean ± SD and compared by multiple unpaired T-test: ***P≤0.001, ****P≤0.0001, n = 3).

As *Zip88E*^*Δ/Δ*^ animals survived to adulthood with no obvious morphological defects, the sensitivity of these mutants to alterations in dietary zinc content was tested. Early first instar larvae of various genotypes were transferred onto *Drosophila* media supplemented with either ZnCl_2_ or the zinc chelator TPEN and survival to adulthood assessed. Compared to the *w*^*1118*^ control strain, survival of the *Zip88E*^*Δ/Δ*^ homozygotes was significantly reduced on both 4 and 8 mmol l^-1^ ZnCl_2_-supplemented food, but was unaffected on lower zinc concentrations and on zinc-chelated media (Fig 2).

**Fig 3:**
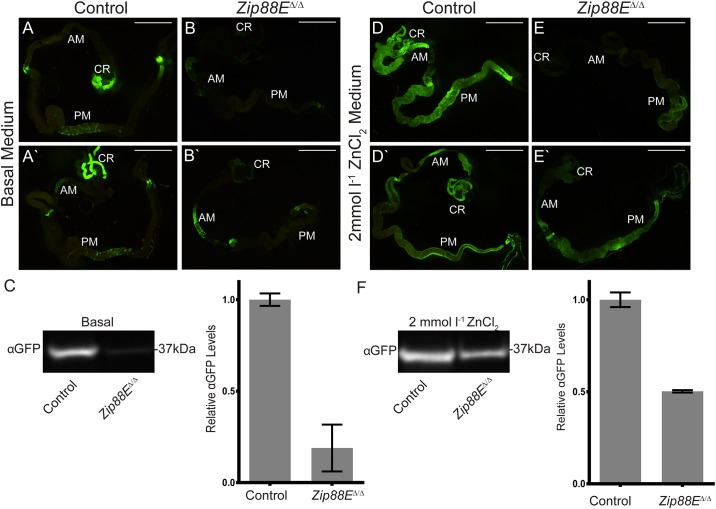
*Zip88E*^*Δ/Δ*^ larvae have decreased *MtnB*:*EYFP* expression. A, B, D, E) *MtnB*:*EYFP* expression in third instar larval midguts from control and *Zip88E*^*Δ/Δ*^ homozygous larvae on basal medium (A and B) and after exposure to 2 mmol l^-1^ ZnCl_2_-supplemented medium (D, E). Variable *MtnB*:*EYFP* expression can be observed between individual flies, therefore an example of both ‘weak’ (top panel) and ‘strong’ (bottom panel) expression has been shown for each genotype and treatment. Decreased *MtnB*:*EYFP* expression is observed in *Zip88E*^*Δ/Δ*^ midguts (B, B’, E and E’) compared to control flies (A, A’, D and D’) on both food types. Fluorescence was observed under dissecting microscope, images were taken with 3 second exposure. N≥10. CR—Crop, AM—Anterior midgut, PM—Posterior midgut. Scale bars represent 1mm. C and F) Representative α-GFP western blots with protein lysate extracted from whole third instar larvae raised on basal (C) and 2 mmol l^-1^ ZnCl_2_ (F) media. Equivalent protein loading was demonstrated by Ponceau S staining (S4 Fig). A single band was detected at 37kDa (see S4 Fig for entire blot), equivalent to the predicted size of the GFP protein produced by *MtnB*:*EYFP*. Band intensity for *Zip88E*^*Δ/Δ*^ larvae was calculated relative to controls and averaged over multiple western blots (values are represented as the mean ± SD, N ≥ 3). C) There is an approximate 5 fold decrease in *MtnB*:*EYFP* expression in *Zip88E*^*Δ/Δ*^ larvae compared to controls when raised on basal media. F) There is an approximate 2 fold decrease in *MtnB*:*EYFP* expression in *Zip88E*^*Δ/Δ*^ larvae compared to controls when exposed to 2 mmol l^-1^ ZnCl_2_-supplemented media. Note that because we are relying on Ponceau S staining as a loading control, these fold changes in band intensity are indicative only and cannot be statistically tested.

To further assess the impact of loss of *Zip88E* on zinc levels in the fly, a reporter gene for the zinc-responsive *Metallothionein B* (*MtnB*) gene was employed to estimate zinc levels in the gastrointestinal tract. *Mtns A* to *D* have all been found to be transcriptionally activated by zinc, copper and cadmium in the fly midgut [12]. *MtnB*:*EYFP* has enhanced Yellow Fluorescent Protein (EYFP) expression driven by the *MtnB* regulatory region [13] and is strongly induced in the midgut by increased dietary zinc content [3, 6, 13]. While basal MtnB:EYFP expression was observed in control third instar larvae (Fig 3A), particularly in the crop / gastric caeca, middle midgut and posterior midgut regions, a strong overall reduction in MtnB:EYFP signal was seen in *Zip88E*^*Δ/Δ*^ mutant larvae (Fig 3B). Quantification of MtnB:EYFP levels by α-GFP western blot from lysates extracted from whole third instar larvae confirmed a dramatic drop in MtnB:EYFP in the mutant larvae (Fig 3C and S4 Fig), particularly in the crop and posterior midgut regions. Induction by 24 hour exposure to 2 mmol l^-1^ ZnCl_2_-supplemented food greatly stimulated MtnB:EYFP in control larvae as expected (Fig 3D) but had considerably lower impact on the reporter in the *Zip88E*^*Δ/Δ*^ mutant larvae (Fig 3E and 3F). Additional midgut images are provided in S5 Fig. Semi-quantitative RT-PCR carried out on other *Zip* genes showed that none of the tested genes were up or down regulated in the *Zip88E*^*Δ/Δ*^ larval midgut (S3 Fig), suggesting that adequate zinc levels are retained in the mutant midgut cells.

**Fig 4:**
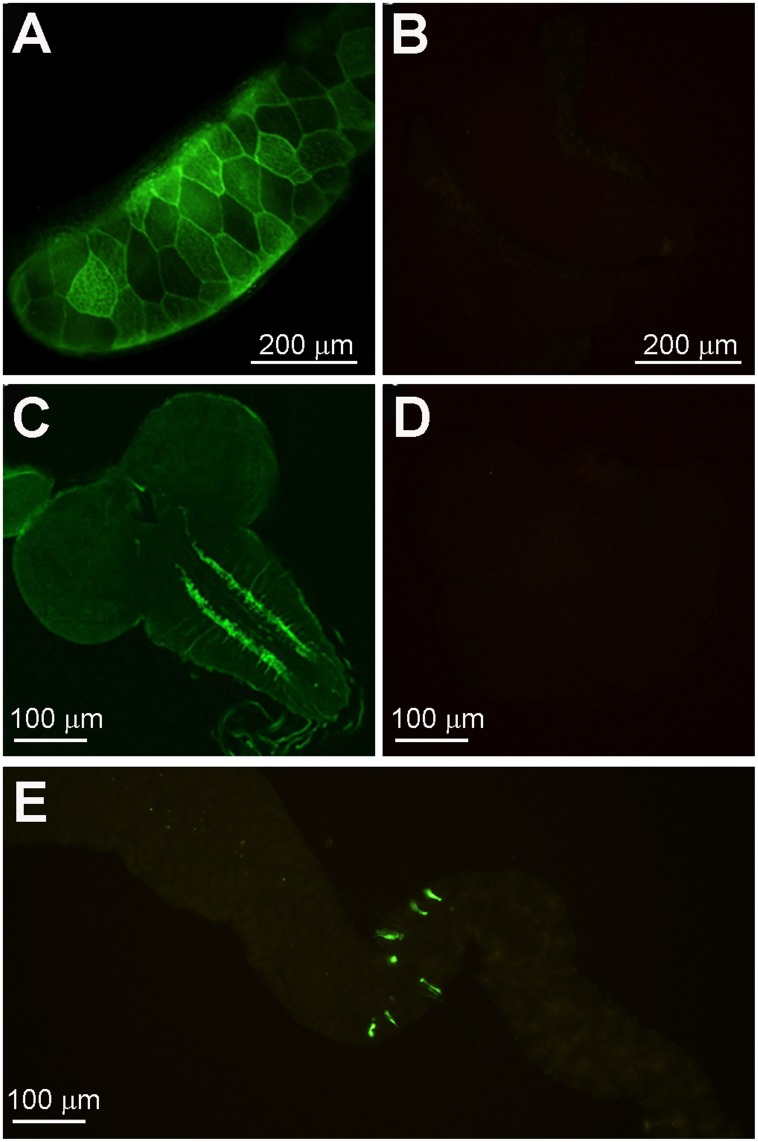
Expression of a *Zip88E* reporter gene is highly restricted. Expression of membrane-bound mCD8::eGFP driven by *Zip88E-GAL4* in third instar larval tissues. GFP expression was observed in the salivary glands (A, B = negative control), in the ventral ganglion of the central nervous system (C, D = negative control) and in a small number of enteroendocrine cells just anterior to the copper cell region of the midgut (E). All images were recorded on a compound fluorescence microscope at 10x (A, B, E)) or 20x (C, D) magnification. In A-D), larvae were fixed and immune-stained with an α-GFP antibody followed by an FITC-conjugated secondary antibody. In E), native GFP signal without antibody staining was imaged.

To determine the endogenous expression pattern of *Zip88E*, a transgenic reporter line, *Zip88E-GAL4*, was generated by cloning the putative upstream enhancer sequences of *Zip88E* in front of the *GAL4* coding sequence. Using *UAS-mCD8*::*GFP* (encoding membrane-bound GFP) in combination with *Zip88E-GAL4*, GFP expression was only observed in three larval tissues, the salivary glands (Fig 4A), in two parallel stripes down the ventral ganglion of the central nervous system (CNS, Fig 4C), and in a collection of enteroendocrine-like cells just anterior to the copper cells of the midgut (Fig 4E). Raising *Zip88E>mCD8*::*GFP* larvae on either ZnCl_2_ or TPEN-supplemented food had no impact on GFP expression, indicating that this gene is not subjected to transcriptional regulation by dietary zinc content (S6 Fig).

**Fig 5:**
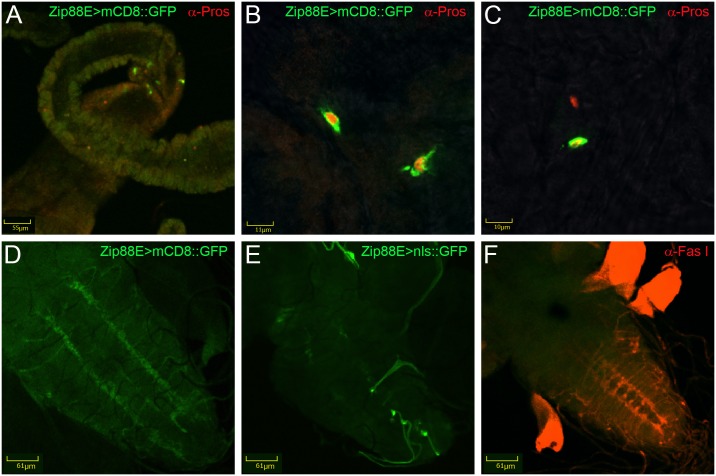
Cells expressing the *Zip88E* reporter gene are enteroendocrine cells. Confocal microscopy of midguts (A-C) and CNS (D-F) of third instar larvae. A-C) Low (A) and high (B, C) magnification images of larval midguts showing Zip88E>mCD8::GFP (green) and α-Prospero (red). While all GFP +ve cells also show nuclear α-Prospero staining, indicating they are enteroendocrine cells, numerous other α-Prospero +ve cells in the same midgut region show no GFP signal. D-F) Larval CNS showing Zip88E>mCD8::GFP (D), Zip88E>nls::GFP (E) and α-Fasciclin I (F, red). The GFP positive axons highlighted by the *Zip88E* reporter gene do not have cell bodies within the CNS and do not appear to lie in the same region as the Fasciclin I +ve axons.

To further explore the origin of the GFP expressed under *Zip88E-GAL4* control, confocal microscopy was performed on larval brains and midguts. Co-staining of *Zip88E>mCD8*::*GFP* midguts with an α-Prospero antibody revealed that all GFP +ve cells in the anterior larval midgut were also Prospero +ve, indicating these *Zip88E*-expressing cells are enteroendocrine cells (Fig 5A–5C). As no cell bodies were visible in the larval ventral ganglion (Fig 4C), the membrane-localised mCD8::GFP reporter was compared to a predominantly nuclear GFP reporter (nls::GFP, Fig 5D and 5E). Whereas the membrane-bound GFP highlighted the parallel stripes down the ventral ganglion as well as lateral projections emanating from these stripes (Fig 5D), the nuclear GFP was observed mainly in a small number of cells at the midline of the ventral ganglion (Fig 5E). While double-staining was not possible due to the presence of the *3xP3*:*DsRed* transgene at the *Zip88E-GAL4* docking site, α-Fasciclin I staining was carried out on control larval brains to provide a morphological landmark. The parallel stripes of Fasciclin I +ve cells appeared to be closer to the ventral ganglion midline than the *Zip88E>mCD8*::*GFP* staining (Fig 5F), therefore the *Zip88E-*expressing sensory neurons are unlikely to be associated with the Fasciclin-expressing dopaminergic neurons.

**Fig 6:**
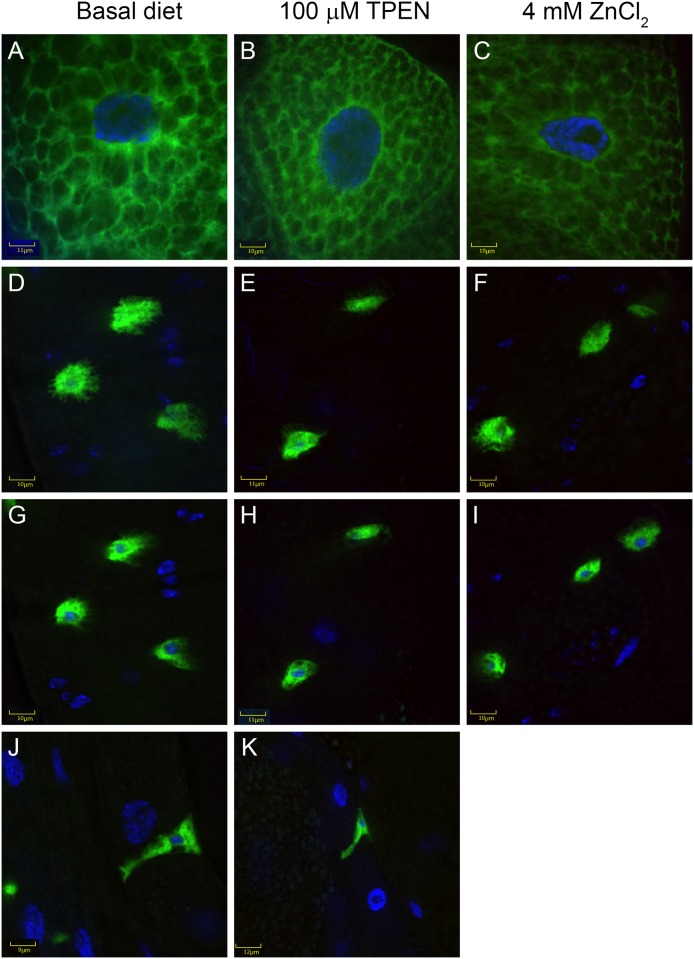
Zip88E protein localisation is not affected by altered zinc dietary content. Confocal microscopy showing salivary gland cells (A-C) and midgut enteroendocrine cells (D-K) with Zip88E-GAL4>Zip88E::eGFP (green) and nuclear DAPI staining (blue) from third instar larvae raised on basal media (A, D, G, J) or media supplemented with 100 μmol l^-1^ TPEN (low zinc, B, E, H, K) or 4 mmol l^-1^ ZnCl_2_ (high zinc, C, F, I). In the enteroendocrine cells, Zip88E::eGFP is observed at both the outer cell surface (D-F) and surrounding the nucleus within the body of the cell (G-I). A lateral view of these cells (J, K) illustrates how they span the width of the midgut epithelial layer.

Previously we have reported that a Zip88E::eGFP fusion protein localises predominantly to an endomembrane, but not the Golgi or ER, when over expressed in larval salivary gland cells [11]. To investigate whether Zip88E is influenced by dietary zinc content, we examined Zip88E::eGFP localisation in the *Zip88E-GAL4* expression domain. The same endomembrane localisation as previously reported was observed in salivary gland cells of larvae raised on normal, low or high-zinc diets (Fig 6A–6C). In the midgut enteroendocrine cells, Zip88E::eGFP was observed throughout the cytosol and on the outer membrane and this localisation did not differ in larvae raised on low or high zinc diets (Fig 6D–6K).

**Fig 7:**
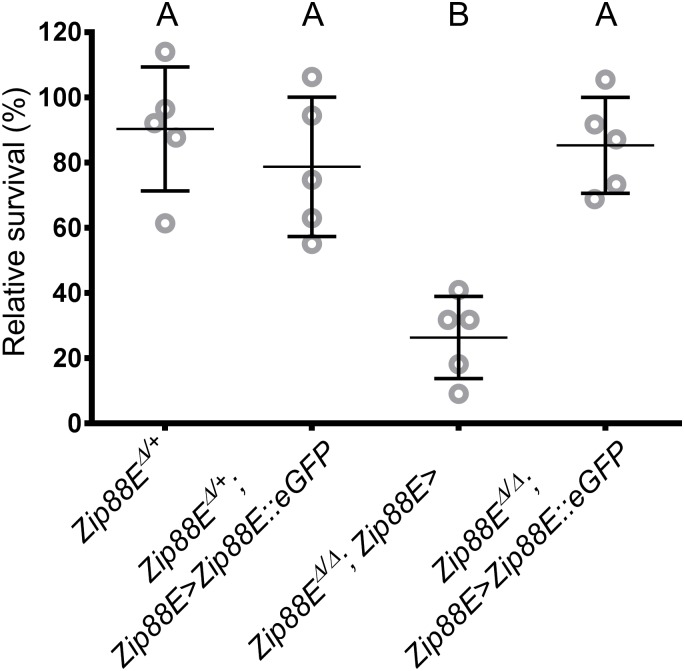
*Zip88E* expression in the *Zip88E-GAL4* expression domain restores dietary zinc tolerance to *Zip88E*^*Δ/Δ*^ mutants. Mean survival rate of flies raised from first instar larvae on 4 mmol l^-1^ ZnCl_2_-supplemented media, relative to survival of the same genotype on basal media. Over expression of *Zip88E*::*eGFP* driven by *Zip88E-GAL4* results in a significant increase in survival of *Zip88E*^*Δ/Δ*^ mutants, back to levels comparable with heterozygote controls. Expression of *Zip88E*::*eGFP* transgene in heterozygotes had no effect on zinc tolerance. Values are represented as the mean survival relative to survival on basal media ± SD. Means with the same letter are not significantly different. Means with different letters (i.e. A and B) are significantly different (P≤0.05, Two-way ANOVA, n = 3).

The highly restricted expression pattern of the *Zip88E-GAL4* reporter gene came as a surprise given the strong zinc-sensitivity phenotype of the *Zip88E*^*Δ/Δ*^ mutants. To examine whether *Zip88E* expression in just these tissues was sufficient to restore systemic *Zip88E* function, survival of *Zip88E*^*Δ/Δ*^ mutants on a high zinc diet was compared with and without the presence of a *Zip88E-GAL4>Zip88E*::*eGFP* transgene combination. Expression of *Zip88E* under the control of the *Zip88E-GAL4* driver completely restored the tolerance of *Zip88E*^*Δ/Δ*^ homozygotes back to wild type levels when larvae were raised on 4 mmol l^-1^ ZnCl_2_-supplemented media (Fig 7). All genotypes tested contained the same *w*^*1118*^ X chromosomes and all but *Zip88E*^*Δ/+*^ had at least one autosomal *mini-white* transgene, abrogating the potential effect of *white* gene presence / absence on zinc content [14].

## Discussion

The strong amino acid sequence conservation between mammalian Zips 1 to 3 and *Drosophila* Zips 42C.1, 42C.2, 89B and 88E suggests that these proteins may be playing similar, possibly overlapping roles in zinc transport. Expression data and functional analysis supports this notion for several of these transporters. *Zips 42C*.*1* and *42C*.*2* have complementary roles in zinc absorption through the iron cells of the fly larval midgut and double knockdown of these transporters causes severe zinc deficiency under zinc-depleted conditions [4]. However, the double knockdown flies are viable under normal dietary conditions suggesting alternative absorption pathways are available. *Zip89B* is expressed both in the iron cells and extensively in the posterior midgut and in mutant larvae lacking this transporter, *Zips 42C*.*1* and *42C*.*1* are upregulated [5], indicating that *Zip89B* may be providing additional zinc uptake capacity that is normally not essential.

This work investigated whether *Zip88E* may also be playing an auxiliary role in zinc absorption. First we confirmed that when over expressed, *Zip88E* acts in a manner consistent with a role in increasing cytosolic zinc levels, as seen previously [11]. Although ectopic *Zip88E* expression in the eye alone has no effect on eye morphology, when combined with *ZipFoi* over expression (increased zinc uptake) or *ZnT63C* RNAi knockdown (decreased zinc efflux), a flattened eye with disrupted morphology results, suggesting a zinc toxicity leading to cell death. However, the interactions seen between *Zip88E* over expression and *Zip48C* knockdown / *ZnT33D* over expression were contrary to expectations since both these manipulations are predicted to decrease cytosolic zinc levels [11] yet both combine with *Zip88E* expression to cause an apparent zinc toxicity phenotype. Rather than a simple cellular zinc uptake role, Zip88E appears to provide a more complex contribution to cellular zinc distribution, consistent with its predominantly intracellular localisation [11]. One caveat in these over expression studies is that Zip88E is being expressed in tissues it may not normally be active in and therefore the interactions observed may not reflect endogenous interactions.

The sensitivity of the *Zip88E*^*Δ/Δ*^ mutant larvae to normally sub-lethal levels of dietary ZnCl_2_ argues against a function in dietary zinc absorption. If *Zip88E* were required normally for zinc uptake in the midgut, the mutants might be expected to show increased sensitivity to zinc depletion or higher tolerance to zinc toxicity, neither of which was observed. The expression pattern of our *Zip88E*-*GAL4* reporter gene was also not consistent with a role for this gene in zinc absorption. The only midgut expression observed was in a highly restricted set of enteroendocrine cells at the anterior / middle midgut boundary. No expression was seen in the iron cells where Zips 42C.1, 42C.2 and 89B are thought to mediate most zinc absorption, nor in any other region of the midgut. While reporter genes do not necessarily capture the entire expression pattern of a gene, the ability of the *Zip88E-GAL4>Zip88E*::*eGFP* transgene combination to rescue the zinc sensitivity phenotype of the *Zip88E*^*Δ/Δ*^ mutant larvae provides evidence that the *Zip88E-GAL4* reporter is driving expression in the cell types responsible for the mutant phenotype. Previously we have reported that RNAi knockdown of *Zip88E* suppresses zinc toxicity phenotypes caused by eye-specific over expression of *Zip71B* or *ZnT86D* [11], implying that *Zip88E* plays an endogenous role in increasing cytosolic zinc levels in the eye. The absence of *Zip88E-GAL4* expression in the eye may be due to an incomplete reporter gene or may indicate that the original RNAi result was in fact due to off-target knockdown of a similar *Zip* such as *Zip42C*.*1*.

The highly restricted expression pattern of the *Zip88E* reporter gene is strongly suggestive of an enteroendocrine role for this gene. In the midgut, we observed expression only in ~six Prospero +ve cells located at the boundary between the anterior and middle midgut segments. Previously, cells in this location have been show to express both allatostatin B / MIP and diuretic hormone DH31 [15]. Allatostatin B is a myo-inhibitory peptide that is able to suppress gut peristalsis in insects. DH31 signals through a G protein-coupled receptor encoded by CG17415 expressed in the Malpighian tubules [16] to regulate fluid secretion but is also able to increase peristaltic muscle contraction [17]. Additional membrane-bound GFP under *Zip88E-GAL4* control was observed in two parallel stripes down the ventral nerve cord of the larval brain as well as occasional lateral projections. No GFP +ve cell bodies were observed in this region indicating that these may represent peripheral sensory neurons projecting to the central nervous system, a possibility supported by the absence of any nuclear signals when using a predominantly nuclear-localised GFP marker.

How might the zinc sensitivity phenotypes observed in the *Zip88E* mutant relate to endocrine signalling? A simple model would have *Zip88E* required for zinc detoxification specifically in the salivary glands and some enteroendocrine cells of the midgut. In the absence of *Zip88E*, these cell types would be more susceptible to zinc toxicity resulting in a decrease in functionality that is manifested as a general decline in larval survival rate. Alternatively, *Zip88E* may be playing a role in detecting elevated zinc levels and mounting a systemic response to this stress; in the absence of such a signal, the developing larvae would be less able to tolerate higher zinc in the diet. In support of this notion, a non-cell autonomous response to loss of *Zip88E* was observed in the decrease in MtnB:EYFP expression in the midguts of *Zip88E* mutant larvae. *Zip88E* is not expressed in the many midgut cells *MtnB* is active in therefore this transcriptional response is presumably caused by a systemic signal. The decrease in MtnB:EYFP could be explained by an inappropriate release of zinc from the midgut cells into the haemolymph. While normally that zinc would be retained safely in the midgut cells, in the *Zip88E* mutants, the absence of an inhibitory signal would allow the influx of zinc to cause damage to internal organs, resulting in a higher mortality rate.

To date, evidence from mammalian and insect systems has mainly supported a model whereby regulation of dietary zinc absorption occurs cell-autonomously at the level of the intestinal enterocytes, so the concept of a mechanism for detecting systemic zinc levels and inducing a non-autonomous response to modify intestinal absorption must be treated as speculative. However precedence for such a mechanism can be found in both the copper and iron homeostasis systems. For instance, genetically-induced copper deficiency in the mouse heart triggers a non-autonomous release of copper from the liver and upregulation of absorption mechanisms in the intestine [18], while hepcidin, produced in the liver in response to iron loading, is able to inhibit iron absorption through enterocytes by inducing endocytosis of the ferroportin iron exporter [19].

It appears that Zip88E has diverged appreciably in function from its closest homologues. Zip89B, 42C.1 and 42C.2. First, its expression pattern is quite different, a situation similar to the comparison between mammalian Zip2 and its close homologues Zip1 and Zip3. However we do not seen any obvious parallels between the expression patterns of *Zip88E* and *Zip2* and while *Zip2* knockout mice reveal defects under zinc deficiency conditions, the *Zip88E* mutant flies are only affected by zinc toxicity.

*Zip88E* differs from its closest fly homologues at the cellular level as well. Unlike the zinc uptake Zips that are all found at the apical plasma membrane, Zip88E::eGFP was observed mainly on intracellular organelles when ectopically expressed [11]. Furthermore, genetic interaction data, looking at the effect of excess *Zip* expression on phenotypes caused by cellular zinc dysregulation, found that *Zip88E* behaved more like *Drosophila Zips 102B* and *99C* than its closest homologues [11]. The mammalian homologue of Zip102B, Zip9, has recently been shown to act as a non-classical androgen receptor for testosterone [20–24], working together with an inhibitor G protein to activate MAP kinase signalling as well as releasing free zinc from mitochondria and the nucleus [23]. These endocrine links suggest that functionally, Zip88E may be more closely related to Zip102B / Zip9 and that an endocrine function for Zip88E may have arisen independently in the invertebrate lineage.

*Zip88E* plays an important role in protecting *Drosophila* larvae against dietary zinc toxicity and this protective action emanates from a small number of specialised cells. It will be of great interest to determine whether the production / activity of known peptides or their receptors is affected by the loss of *Zip88E*, and whether the *Zip88E* mutant zinc sensitivity phenotype can be replicated by inhibiting such peptide activity. A conclusive demonstration of a systemic zinc sensing / response mechanism would dramatically change our view of how this critical biometal is regulated in animals.

## Supporting information

S1 Table Oligonucleotide sequences of PCR primers used in this study. (DOCX)

S1 Fig Over expression of *Zip88E* shows no interaction with over expression or RNAi knockdown of most other *Drosophila* Zip and *ZnT* genes. *GMR>Zip88E*::*FLAG* in combination with the over expression and RNAi suppression (IR) of all remaining *Zip* / *ZnT* genes not shown in Fig 1. A) *Zip88E*::*FLAG*-only control. B) *GMR-GAL4*-only control. C) *GMR>Zip88E*::*FLAG*. D-H’) *GMR>Zip88E*::*FLAG* together with D) *Zip88E*::*FLAG*, E) *Zip42C*.*2*::*FLAG*, F) *Zip89B*::*FLAG*, G) *Zip99C*::*FLAG*, H) *ZipCatsup*::*FLAG*, I) *Zip71B*::*eGFP*, J) *Zip102B*, K) *Zip48C*, L) *ZnT41F*::*FLAG*, M) *ZnT63C*::*FLAG*, N) *ZnT35C*::*FLAG*, O) *ZnT77C*::*FLAG*, P) *ZnT86D*::*FLAG*, Q) *Zip88E IR(1)*, R) *Zip88E IR(2)*, S) *Zip42C*.*1 IR*, T) *Zip42C*.*2 IR*, U) *Zip89B IR*, V) *ZipFoi IR*, W) *Zip99C IR*, X) *ZipCatsup IR*, Y) *Zip71B IR*, Z) *Zip102B IR(1)*, A’) *Zip102B IR(2)*, B’) *ZnT41F IR*, C’) *ZnT33D IR*, D’) *ZnT35C IR(1)*, E’) *ZnT35C IR(2)*, F’) *ZnT77C IR*, G’) *ZnT86D IR* and H’) *ZnT49B IR*.
(TIF)

S2 Fig Generation of a putative null deletion allele of *Zip88E* by imprecise P-element excision. A) Annotated schematic of the *Zip88E* genomic region showing the 5’ and 3’ untranslated regions (UTRs), exons 1 to 4, the location of the original P(EPgy2) element in the 5’ UTR and the location of the oligonucleotide primers used to screen for internal deletions caused by mobilisation of the P(EPgy2) element. B) Schematic of the *Zip88E* region after a precise P(EPgy2) excision event. C) Schematic of the *Zip88E* region after an imprecise P(EPgy2) excision event that deleted all of the 5’UTR and exons 1 and 2 of *Zip88E*, resulting in a putative null allele. This allele, called *Zip88E*^*Δ*^, was used in all functional analyses presented here. D) Annotated alignment of the *Zip88E* genomic sequence from control (top line) and *Zip88E*^*Δ/Δ*^ (bottom line) flies, showing the full extent of the *Zip88E* deletion. E) Agarose gel showing PCR products generated using primers *Zip88E* PF1 and PR2 on gDNA extracted from single adult flies of genotypes *Zip88E*^*Δ/Δ*^ (1–5) or *w*^*1118*^ control (6–10). All mutant flies show a ~800 bp PCR product compared to the ~1500 bp product present in the control flies.
(TIF)

S3 Fig Semi-quantitative PCR of *Zip* gene expression in larval midguts. A) 2.5% agarose gels showing PCR products from cDNA generated from mRNA extracted from dissected midguts of: *w*^*1118*^ control (1); *Zip89B* ^*Δ/Δ*^ (2); and *Zip88E*^*Δ Δ/Δ*^ (3) third instar larvae. Products for *Zip88E*, *Zip89B*, *ZipFoi*, *Zip99C*, *ZipCatsup*; *Zip71B*, *Zip102B*, *Zip48C* and *RP49* are seen for each genotype. *Zip42C*.*1* and *Zip42C*.*2* did not produce bands of sufficient intensity for analysis. The lower molecular weight band seen for *Zip71B* is non-specific. Results shown are representative of two independent cDNA extractions / PCR analyses. B) Separated scatter plot showing quantification of PCR product band intensities from gels illustrated in A (n = 2). Band intensities for each *Zip* gene were determined using ImageJ then normalised to the control gene (*RP49*) band intensities for that particular cDNA sample. For each *Zip* gene, the normalised band intensity from the *w*^*1118*^ control cDNA sample was set at 1 then band intensities of the two mutant cDNA samples are expressed relative to the control. This semi-quantitative gene expression analysis indicates that no *Zip88E* expression was detectable in the *Zip88E*^*Δ/Δ*^ mutant larvae and no *Zip89B* expression was detectable in the *Zip89B*^*Δ/Δ*^ mutant larvae, confirming that these two mutations are most likely null mutations. While no *Zip* genes showed altered expression levels in *Zip88E*^*Δ/Δ*^ mutant midguts, *Zip88E*, *ZipFoi*, *ZipCatsup*, *Zip102B* and *Zip48C* all appeared to be down-regulated in *Zip89B*^*Δ/Δ*^ mutant midguts. *Zip71B* could not be analysed due to the presence of non-specific PCR products.
(TIF)

S4 Fig Equivalent sample loading for westerns blots as shown by Ponceau S staining. A) α-GFP western blot on lysates from either *w*^*1118*^ or *Zip88E*^*Δ/Δ*^ whole larvae both containing the *MtnB*:*EYFP* transgene, raised on either basal medium or medium supplemented with 2 mmol l^-1^ ZnCl_2_. Two replicates are shown for each condition. A strong GFP signal is observed at molecular mass of ~37kDa. The GFP signal is more intense with *w*^*1118*^ than *Zip88E*^*Δ/Δ*^ larvae and is induced by exposure to high dietary zinc. B) Ponceau S staining of the membrane blotted in (A). Similar Ponceau S intensity is seen in each lane indicating that roughly equal amounts of protein are being loaded in each lane.
(TIF)

S5 Fig Additional images of *MtnB*:*EYFP* expression in the larval midgut. *MtnB*:*EYFP* expression in third instar larval midguts from *w*^*1118*^ control (A and B) and *Zip88E*^*Δ/Δ*^ homozygous larvae (C, D) on basal medium (A, C) and after exposure to 2 mmol l^-1^ ZnCl_2_-supplemented medium (B, D). Variable *MtnB*:*EYFP* expression can be observed between individual flies but overall, decreased *MtnB*:*EYFP* expression is observed in *Zip88E*^*Δ/Δ*^ midguts compared to control flies on both food types. Fluorescence was observed under dissecting microscope, images were taken with 3 second exposure.
(TIF)

S6 Fig The *Zip88E-GAL4* reporter gene does not respond to changes in dietary zinc content. Confocal microscopy showing dissected third larval instar salivary glands (A-C), midguts (D-F) and CNS (G-I) from larvae containing either *Zip88E>nls*::*GFP* (A-C) or *Zip88E>mCD8*::*GFP* (D-I) reporter gene combinations. Larvae were raised on basal medium (A, D, G) or media supplemented with 100 μmol l^-1^ TPEN (low zinc, B, E, H) or 4 mmol l^-1^ ZnCl_2_ (high zinc, C, F, I). No changes in the overall *Zip88E-GAL4* expression pattern were observed on either low or high zinc diets compared to basal medium. Native GFP signal (without α-GFP antibody staining) is shown in each case and images are representative of >10 individuals for each diet.
(TIF)

## Abbreviations

(e)GFP: (enhanced) green fluorescent protein
TPEN: N,N,N’,N’-tetrakis (2-pyridylmethyl)-ethylenedidiamine
