# Supplementary material for: A role for the Drosophila zinc transporter Zip88E in protecting against dietary zinc toxicity
Source: PLoS One. 2017 Jul 13;12(7):e0181237. doi: 10.1371/journal.pone.0181237 (PMC5509326; doi:10.1371/journal.pone.0181237)
Supplement: S1 Table — (DOCX) [file pone.0181237.s001.docx]

**S1 Table. Oligonucleotide sequences of PCR primers used in this study.**

| **Primer name** | **Sequence** |
| --- | --- |
| Zip88E RT F | TCACATGGGCATTCGCACCAT |
| Zip88E RT R | GGCGTCCTCTACACTGGCA |
| *Zip88E* pro F1 | CCGAAGCTTCCTAGGCTCGAGGCATGCCATGTTGCGCGGGTAGACGC |
| *Zip88E* pro R1 | GAGGTACCTGTATCAACTGCCAGACTGT |
| *Zip88E* PF1 | AAACTGCTGAGGTTGGCCAACTTCG |
| *Zip88E* PR1 | CTTTTATCAGCGGAACCCAATACGT |
| *Zip88E* PR2 | TCGGCATTTCGGTGTTTGCC |
| *Zip89B RT F* | ATGTTTGGCCTGCAGCAGTT |
| *Zip89B RT R* | TCTCTTGGTGGTTGTGTGTGTGT |
| *Zip99C RT F* | TGGATCCTTTTTGGTGTCCTTCAGA |
| *Zip99C RT R* | AGCGACTGAATCCGGATCGAA |
| *Zip48C RT F* | TAACAGCACCAACATGATGATCCAGA |
| *Zip48C RT R* | ACTCCATTGCGCTTAGAGTCCT |
| *ZipFoi RT F* | GTGGCTGCGGGTCTGTTC |
| *ZipFoi RT R* | TTTGTGCGAGGCCGAGAT |
| *Zip71B RT F* | ATGACCCCAAGCACCGACAT |
| *Zip71B RT R* | ATGTTTTTGTGGAGATCGTTGTGGTT |
| *Zip102B RT F* | TCAACGTCTTGATGGCTAGTGGT |
| *Zip102B RT R* | AAAACAATGCCACACTAACTCTTGGATT |
| *ZipCatsup RT F* | TGAAAGGCACACCAAGGCAAA |
| *ZipCatsup RT R* | AGCGGCACTGATAAGAAGAGTTGA |
| *RP49 F* | AAGAAGCGCACCAAGCACTTCATC |
| *RP49 R* | TCTGTTGTCGATACCCTTGGGCTT |
