# Supplementary figures and images for: A role for the Drosophila zinc transporter Zip88E in protecting against dietary zinc toxicity
Source: PLoS One. 2017 Jul 13;12(7):e0181237. doi: 10.1371/journal.pone.0181237 (PMC5509326; doi:10.1371/journal.pone.0181237)

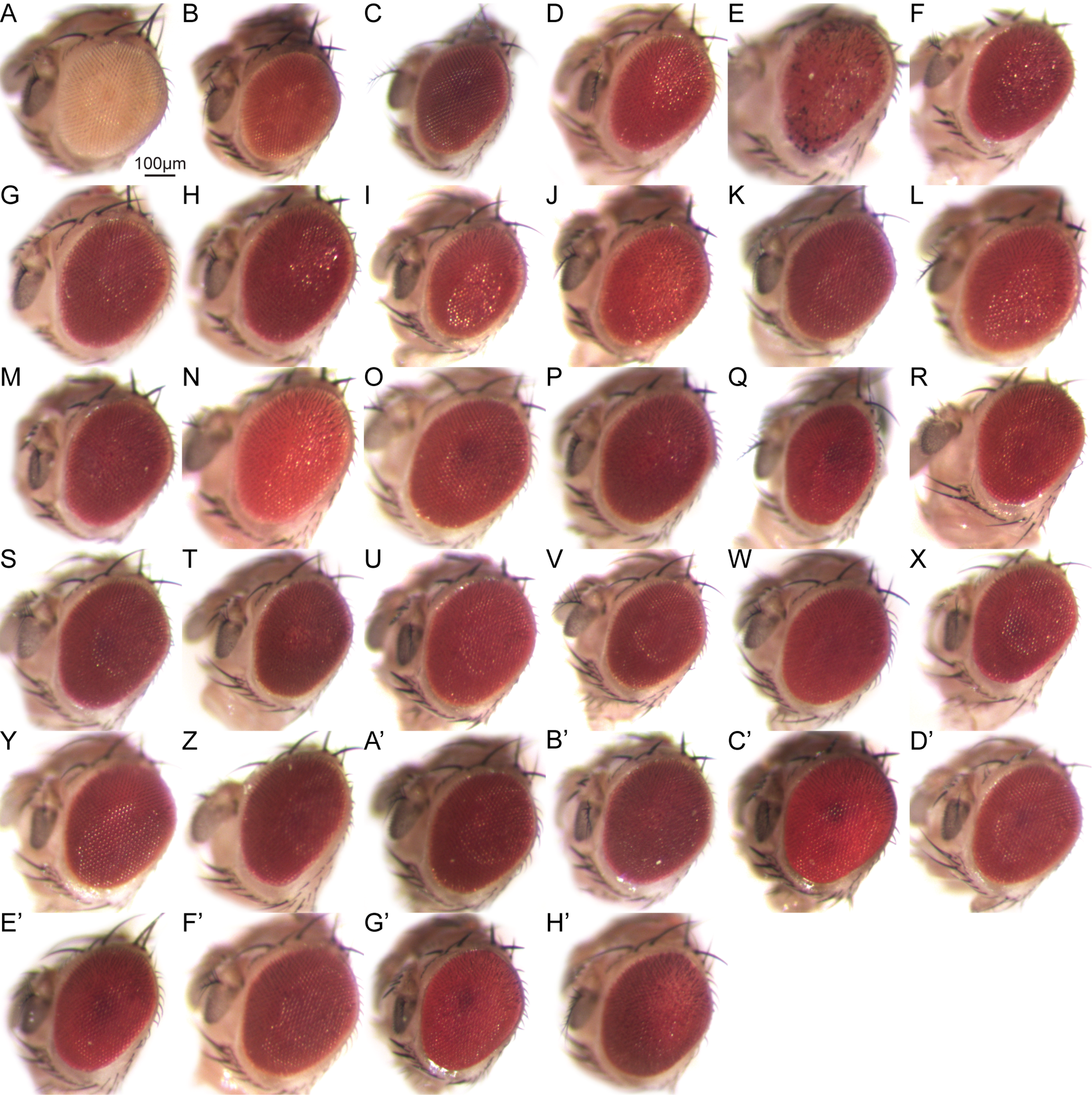

Supplement: S1 Fig — GMR>Zip88E::FLAG in combination with the over expression and RNAi suppression (IR) of all remaining Zip / ZnT genes not shown in Fig 1. A) Zip88E::FLAG-only control. B) GMR-GAL4-only control. C) GMR>Zip88E::FLAG. D-H’) GMR>Zip88E::FLAG together with D) Zip88E::FLAG, E) Zip42C.2::FLAG, F) Zip89B::FLAG, G) Zip99C::FLAG, H) ZipCatsup::FLAG, I) Zip71B::eGFP, J) Zip102B, K) Zip48C, L) ZnT41F::FLAG, M) ZnT63C::FLAG, N) ZnT35C::FLAG, O) ZnT77C::FLAG, P) ZnT86D::FLAG, Q) Zip88E IR(1), R) Zip88E IR(2), S) Zip42C.1 IR, T) Zip42C.2 IR, U) Zip89B IR, V) ZipFoi IR, W) Zip99C IR, X) ZipCatsup IR, Y) Zip71B IR, Z) Zip102B IR(1), A’) Zip102B IR(2), B’) ZnT41F IR, C’) ZnT33D IR, D’) ZnT35C IR(1), E’) ZnT35C IR(2), F’) ZnT77C IR, G’) ZnT86D IR and H’) ZnT49B IR. (TIF) [file pone.0181237.s002.tif]

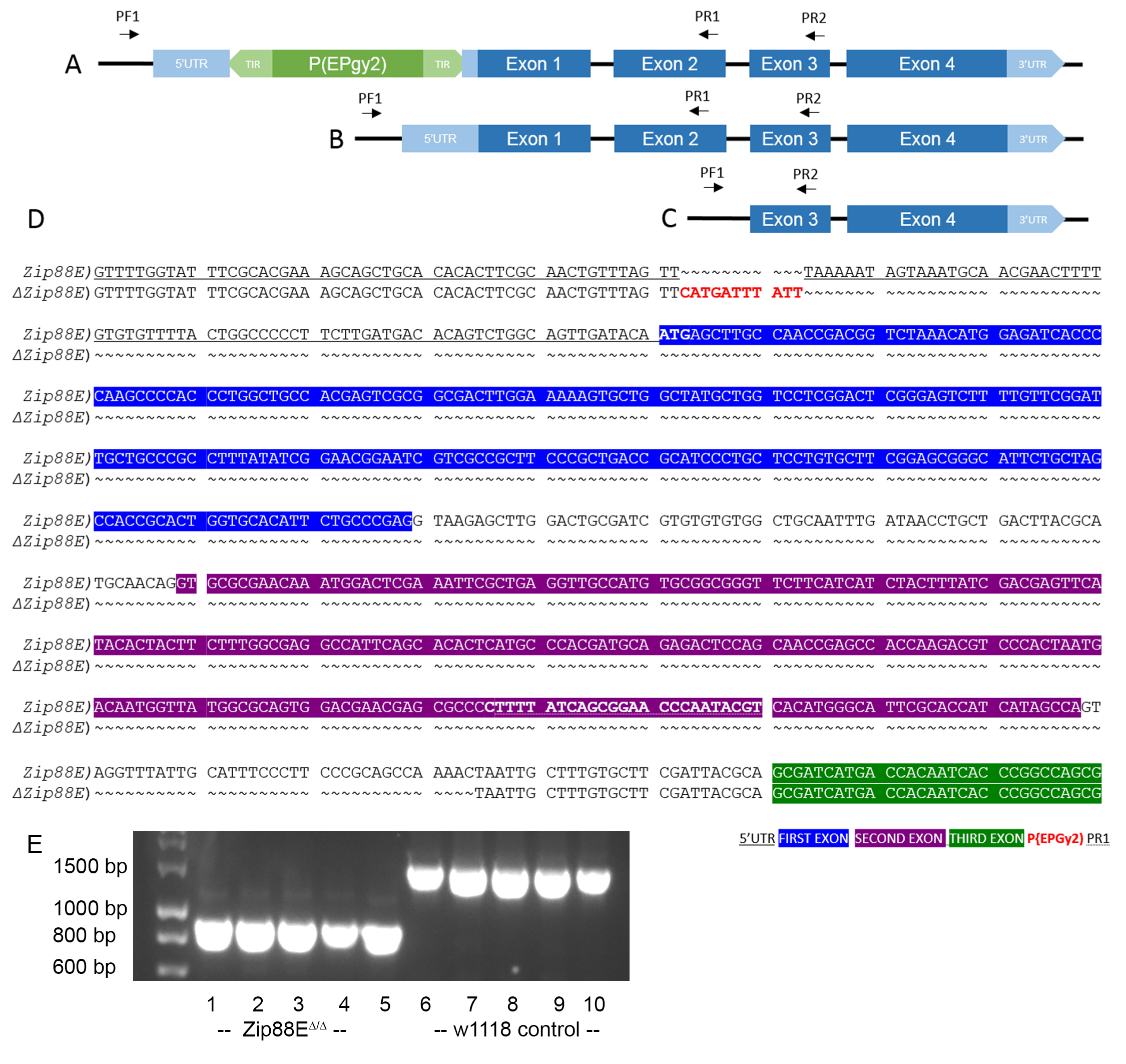

Supplement: S2 Fig — A) Annotated schematic of the Zip88E genomic region showing the 5’ and 3’ untranslated regions (UTRs), exons 1 to 4, the location of the original P(EPgy2) element in the 5’ UTR and the location of the oligonucleotide primers used to screen for internal deletions caused by mobilisation of the P(EPgy2) element. B) Schematic of the Zip88E region after a precise P(EPgy2) excision event. C) Schematic of the Zip88E region after an imprecise P(EPgy2) excision event that deleted all of the 5’UTR and exons 1 and 2 of Zip88E, resulting in a putative null allele. This allele, called Zip88EΔ, was used in all functional analyses presented here. D) Annotated alignment of the Zip88E genomic sequence from control (top line) and Zip88EΔ/Δ (bottom line) flies, showing the full extent of the Zip88E deletion. E) Agarose gel showing PCR products generated using primers Zip88E PF1 and PR2 on gDNA extracted from single adult flies of genotypes Zip88EΔ/Δ (1–5) or w1118 control (6–10). All mutant flies show a ~800 bp PCR product compared to the ~1500 bp product present in the control flies. (TIF) [file pone.0181237.s003.tif]

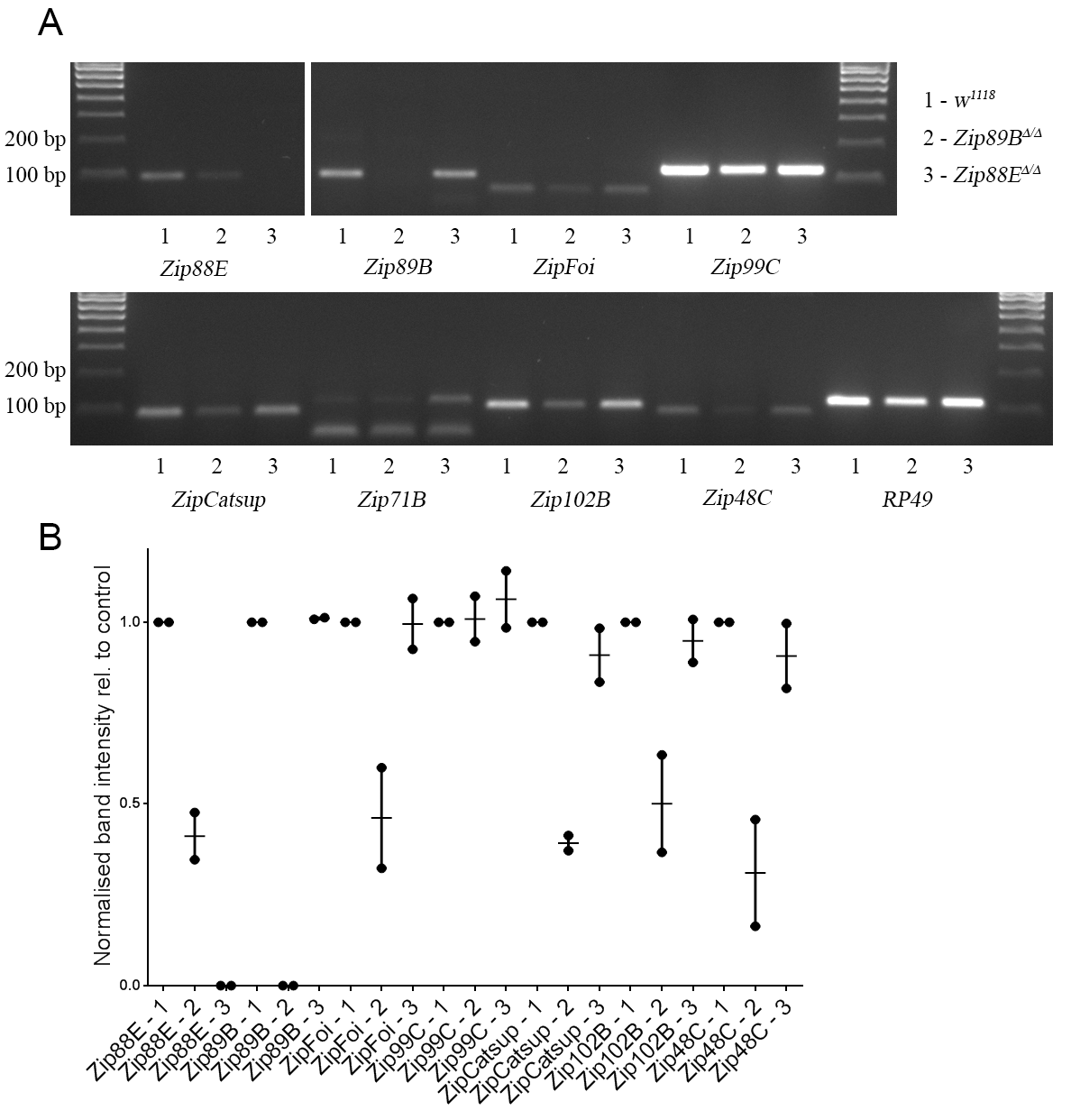

Supplement: S3 Fig — A) 2.5% agarose gels showing PCR products from cDNA generated from mRNA extracted from dissected midguts of: w1118 control (1); Zip89B Δ/Δ (2); and Zip88EΔ Δ/Δ (3) third instar larvae. Products for Zip88E, Zip89B, ZipFoi, Zip99C, ZipCatsup; Zip71B, Zip102B, Zip48C and RP49 are seen for each genotype. Zip42C.1 and Zip42C.2 did not produce bands of sufficient intensity for analysis. The lower molecular weight band seen for Zip71B is non-specific. Results shown are representative of two independent cDNA extractions / PCR analyses. B) Separated scatter plot showing quantification of PCR product band intensities from gels illustrated in A (n = 2). Band intensities for each Zip gene were determined using ImageJ then normalised to the control gene (RP49) band intensities for that particular cDNA sample. For each Zip gene, the normalised band intensity from the w1118 control cDNA sample was set at 1 then band intensities of the two mutant cDNA samples are expressed relative to the control. This semi-quantitative gene expression analysis indicates that no Zip88E expression was detectable in the Zip88EΔ/Δ mutant larvae and no Zip89B expression was detectable in the Zip89BΔ/Δ mutant larvae, confirming that these two mutations are most likely null mutations. While no Zip genes showed altered expression levels in Zip88EΔ/Δ mutant midguts, Zip88E, ZipFoi, ZipCatsup, Zip102B and Zip48C all appeared to be down-regulated in Zip89BΔ/Δ mutant midguts. Zip71B could not be analysed due to the presence of non-specific PCR products. (TIF) [file pone.0181237.s004.tif]

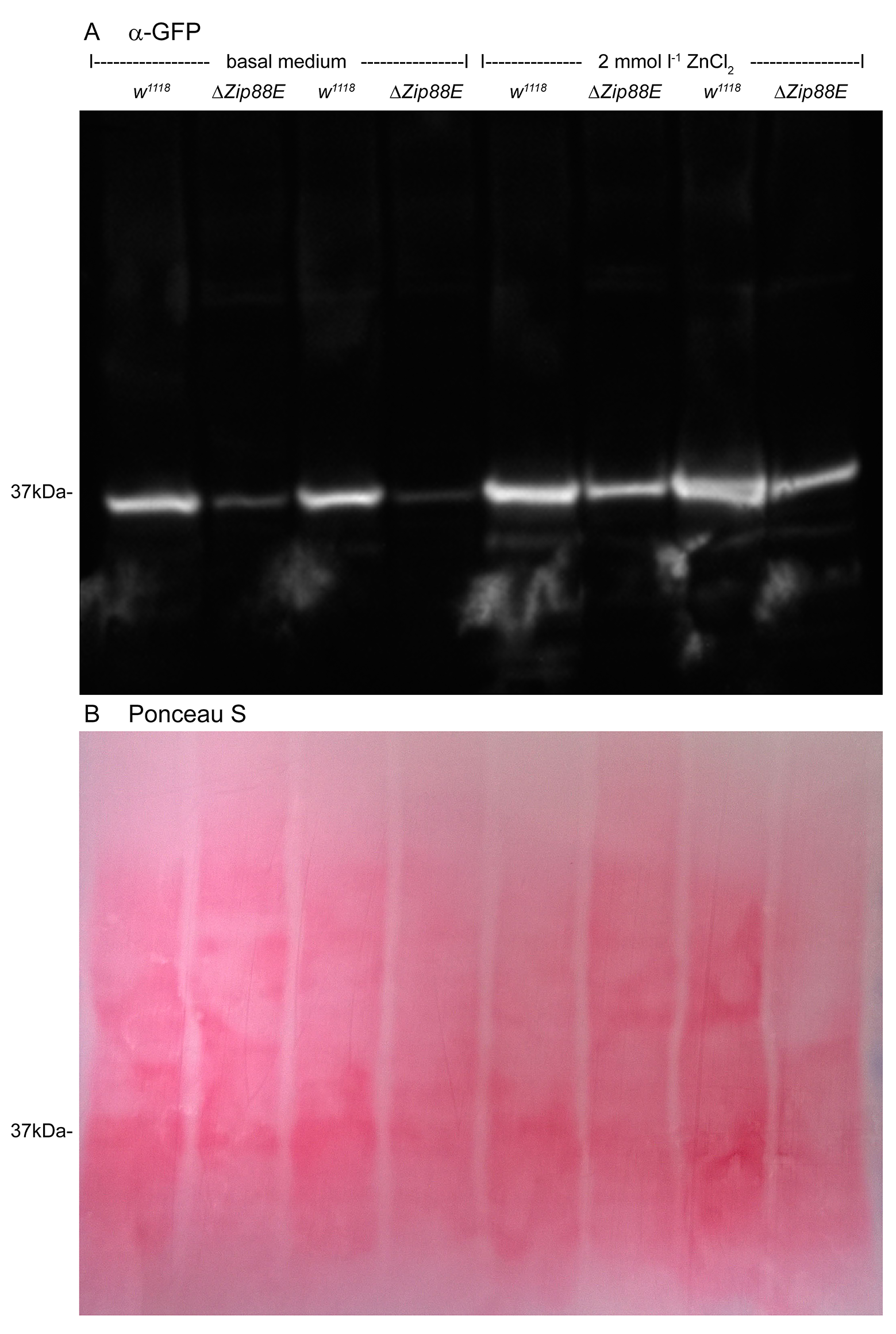

Supplement: S4 Fig — A) α-GFP western blot on lysates from either w1118 or Zip88EΔ/Δ whole larvae both containing the MtnB:EYFP transgene, raised on either basal medium or medium supplemented with 2 mmol l-1 ZnCl2. Two replicates are shown for each condition. A strong GFP signal is observed at molecular mass of ~37kDa. The GFP signal is more intense with w1118 than Zip88EΔ/Δ larvae and is induced by exposure to high dietary zinc. B) Ponceau S staining of the membrane blotted in (A). Similar Ponceau S intensity is seen in each lane indicating that roughly equal amounts of protein are being loaded in each lane. (TIF) [file pone.0181237.s005.tif]

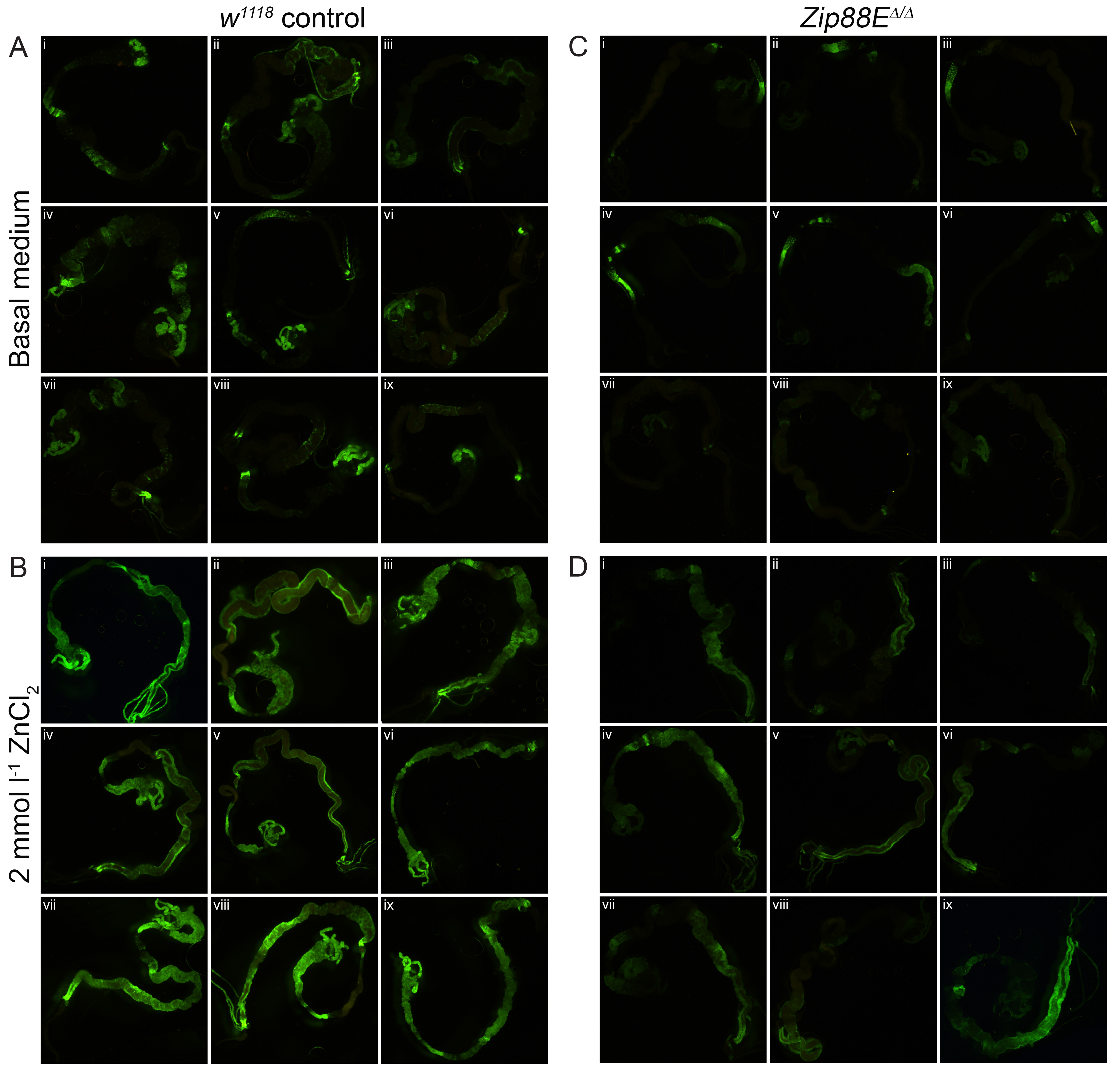

Supplement: S5 Fig — MtnB:EYFP expression in third instar larval midguts from w1118 control (A and B) and Zip88EΔ/Δ homozygous larvae (C, D) on basal medium (A, C) and after exposure to 2 mmol l-1 ZnCl2-supplemented medium (B, D). Variable MtnB:EYFP expression can be observed between individual flies but overall, decreased MtnB:EYFP expression is observed in Zip88EΔ/Δ midguts compared to control flies on both food types. Fluorescence was observed under dissecting microscope, images were taken with 3 second exposure. (TIF) [file pone.0181237.s006.tif]

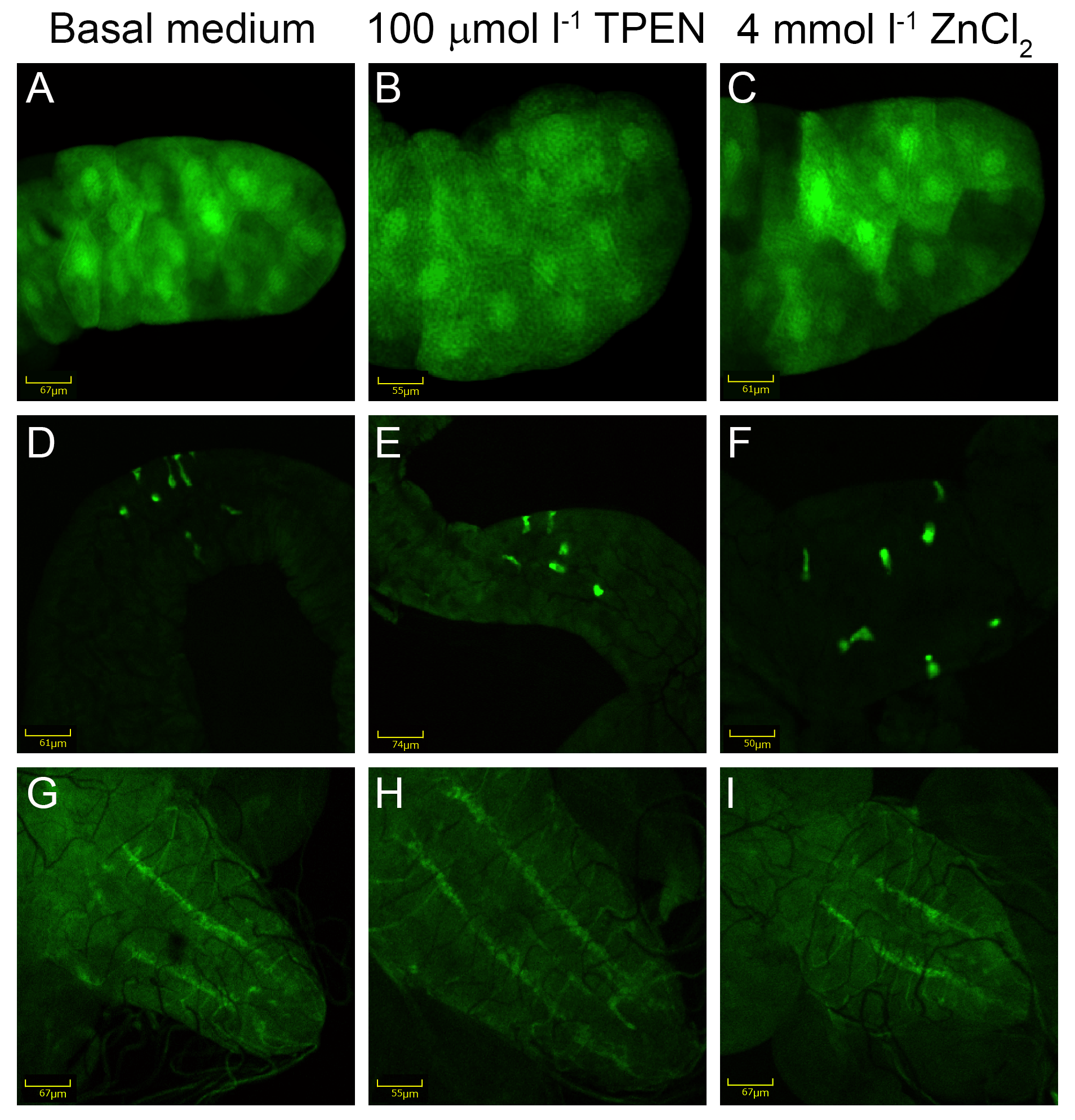

Supplement: S6 Fig — Confocal microscopy showing dissected third larval instar salivary glands (A-C), midguts (D-F) and CNS (G-I) from larvae containing either Zip88E>nls::GFP (A-C) or Zip88E>mCD8::GFP (D-I) reporter gene combinations. Larvae were raised on basal medium (A, D, G) or media supplemented with 100 μmol l-1 TPEN (low zinc, B, E, H) or 4 mmol l-1 ZnCl2 (high zinc, C, F, I). No changes in the overall Zip88E-GAL4 expression pattern were observed on either low or high zinc diets compared to basal medium. Native GFP signal (without α-GFP antibody staining) is shown in each case and images are representative of >10 individuals for each diet. (TIF) [file pone.0181237.s007.tif]
